# Decreased MCM2-6 in Drosophila S2 Cells Does Not Generate Significant DNA Damage or Cause a Marked Increase in Sensitivity to Replication Interference

**DOI:** 10.1371/journal.pone.0027101

**Published:** 2011-11-15

**Authors:** Isabelle Crevel, Gilles Crevel, Thierry Gostan, Christelle de Renty, Vincent Coulon, Sue Cotterill

**Affiliations:** 1 Department Basic Medical Sciences, St. Georges University London, London, United Kingdom; 2 DNA Combing Facility, Institute of Molecular Genetics, CNRS UMR 5535 and University Montpellier 1 and 2, Montpellier, France; University of Minnesota, United States of America

## Abstract

A reduction in the level of some MCM proteins in human cancer cells (MCM5 in U20S cells or MCM3 in Hela cells) causes a rapid increase in the level of DNA damage under normal conditions of cell proliferation and a loss of viability when the cells are subjected to replication interference. Here we show that Drosophila S2 cells do not appear to show the same degree of sensitivity to MCM2-6 reduction. Under normal cell growth conditions a reduction of >95% in the levels of MCM3, 5, and 6 causes no significant short term alteration in the parameters of DNA replication or increase in DNA damage. MCM depleted cells challenged with HU do show a decrease in the density of replication forks compared to cells with normal levels of MCM proteins, but this produces no consistent change in the levels of DNA damage observed. In contrast a comparable reduction of MCM7 levels has marked effects on viability, replication parameters and DNA damage in the absence of HU treatment.

## Introduction

The MCM2-7 proteins form a complex which is a central player in DNA replication in cells (reviewed [1]). It is involved at two stages of the process: initiation, where it is important for the formation of the preRC, and elongation, where it is thought to be the primary helicase which unwinds the DNA ahead of the replication fork.

A striking feature of the MCM complex is that it binds to chromatin at high concentrations relative to the number of origins present and also to the levels of other replication proteins such as ORC [2]–[6]. This has led to the proposal of a number of mechanisms of action for the MCM proteins which involve the action of multiple complexes at each origin [7], [8]. However in Xenopus extracts [9], Drosophila S2 cells [10] and for MCM5 in U20S cells [11] and MCM3 in Hela cells [12] the levels of the MCM proteins can be drastically reduced without suffering an apparent loss in the efficiency of unperturbed DNA replication or cell survival. Several recent studies have led to the hypothesis that one function of the additional MCM proteins maybe in permitting survival after perturbation of DNA replication. Studies in Xenopus extracts [13] showed that if DNA replication was inhibited with aphidicolin and the S phase checkpoint ATR/ATM kinases were inhibited with caffeine then extracts where fewer MCMs had been loaded onto chromatin (due to the addition of geminin) were less proficient at replication. Subsequently studies in human cancer lines were also used to suggest a similar hypothesis. If U2OS cells depleted of MCM5 by RNAi to levels which do not affect their normal replication are challenged with HU they are less able to survive [11]. Hela cells are not able to survive the equivalent MCM5 depletion (or the depletion of MCM4, 6 or 7) however depletion of MCM3 in these cells produced no short term changes in viability or replication initiation and elongation but did seem to result in increased DNA damage, as well as a decreased ability of the cells to survive HU/aphidicolin challenge [12]. The model proposed from these studies is that after replication interference the replication fork restarts again from other normally silent origins. If MCM proteins are limiting this is not possible resulting in decreased replication and cell viability.

In our previous studies with Drosophila S2 cells [10] we showed that, with the exception of MCM7, reduction of any of the members of the MCM complex by >95% had little effect on cell viability or DNA replication under conditions where DNA replication was not perturbed. The data presented here extend those studies. Firstly using more sensitive ways of looking at DNA replication we are still unable to detect significant changes in replication under unperturbed conditions. Secondly, following on from recent studies which suggest that cancer cells respond differently to changes in the levels of other replication proteins eg cdt1 [14], we determined whether S2 cells (which are not transformed) showed the same dependence on high levels of the MCM proteins for viability, DNA replication and DNA damage resistance after replication interference. Using similar techniques to those used for the published studies in human cells we were not able to detect the same striking changes in these parameters in Drosophila S2 cells. This suggests that the requirement for a reservoir of MCM proteins in S2 cells cannot be entirely explained by a role in recovery after DNA replication interference.

## Results

### Reduction of MCM 2-6 in S2 cells does not affect nucleotide incorporation and fork movement

**Figure 1:**
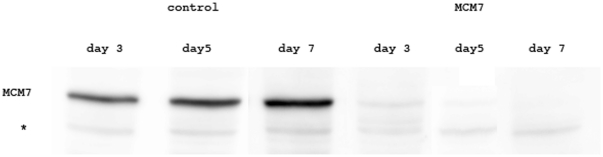
Western blot to demonstrate the depletion of MCM7 from S2 cells by dsRNA interference. The levels of MCM7 protein were determined 3, 5 and 7 days after the addition of dsRNA in control cells and cells treated with dsRNA against MCM7 as shown. Loading was checked by comparison with Ponceau red staining of the blot before development (data not shown). The cross-reacting but unrelated band (*) also provides an indication that loading between the fractions is comparable.

Our previous work showed that dsRNAi depletion of MCM3 reduced MCM3 and MCM5 levels, and MCM6 depletion reduced MCM2, MCM4 and MCM6 levels [10]. Co-depletion of multiple proteins might be expected to have a larger cellular effect. Therefore for the studies presented here we chose to deplete MCM3 and MCM6 together with MCM5 (which only affects MCM5). We also targeted MCM7 (which causes a reduction of MCM4 and MCM7). MCM7 depletion has a drastic effect on cell viability in the absence of DNA damage, we would therefore expect that depletion of this protein should have effects on the parameters which we are looking at, thus allowing it to serve as a positive control. As for our previous studies the depletion of the protein levels for MCM3, MCM5 and MCM6 were all greater than 90%. We did not previously show the equivalent data for MCM7 due to the lack of an efficient antibody. We have therefore generated a new MCM7 antibody and Figure 1 shows that under our standard dsRNA depletion conditions we are able to achieve comparable depletion of MCM7 protein levels.

In our original studies the depletion of MCM3, MCM5 or MCM6 had no apparent effects on cell viability, cell cycle distribution of the cells or PCNA binding to chromatin. To rule out the possibility that MCM depletion caused subtle effects that were not detected using these techniques we re-examined the MCM depleted cells using two techniques which should pick up more subtle effects. - BrdU incorporation and molecular combing.

**Figure 2:**
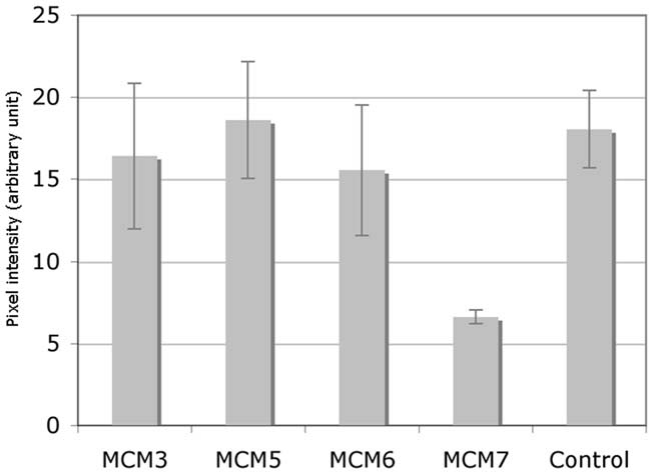
Dot blot analysis to compare BrdU incorporation by MCM depleted and control cells. S2 cells after 5 days of treatment with dsRNAi against MCM3, MCM5 MCM6 MCM7 or a control human fragment were labeled with BrdU for 1 h and the DNA prepared and analysed as described in materials and methods. These data are the average of 2 independent experiments.

We first measured DNA synthesis directly by following the incorporation of BrdU into the DNA. Figure 2 shows that while BrdU incorporation in an MCM7 depleted cell line is markedly reduced, depletion of MCM3, MCM5 and MCM6 had no significant effect on DNA synthesis as measured by this method.

**Figure 3:**
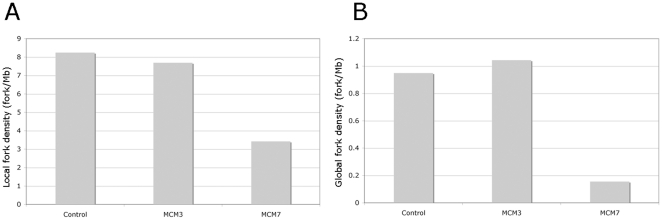
Relative local and global fork densities of MCM depleted and control cells as measured using molecular combing. These data were derived as explained in materials and methods – please also see Figure S1.

We next used DNA molecular combing to measure the origin spacing and rate of fork movement in MCM3, MCM7 and control cells. We measured global fork density (taking into account all observed DNA fibres), local fork density (only taking into account fibres showing incorporation) and the rate of fork movement. The data obtained is shown in Figure 3 and Figure S1. MCM3 depleted cells show no significant difference in global or local origin density. By contrast MCM7 depletion from cells causes a significant change in both global and local origin density. In neither case was a significant change seen in the rate of fork movement (see observed BrdU track lengths in Figure S1).

### Reduction of MCM 2-6 in S2 cells has minimal effects on viability in the presence HU

#### Titration HU levels

To be consistent with earlier studies in Xenopus extracts and human cells the level of replication interference required is one that does not significantly inhibit replication in wild type cells. For human cells the levels used varied between 0.030 mM [12] and 0.2–1.3 mM [11]. To determine the optimal concentration to use for Drosophila, we titrated the amount of HU added to cells to determine a level which only resulted in a transient cell cycle delay in untreated S2 cells.

**Figure 4:**
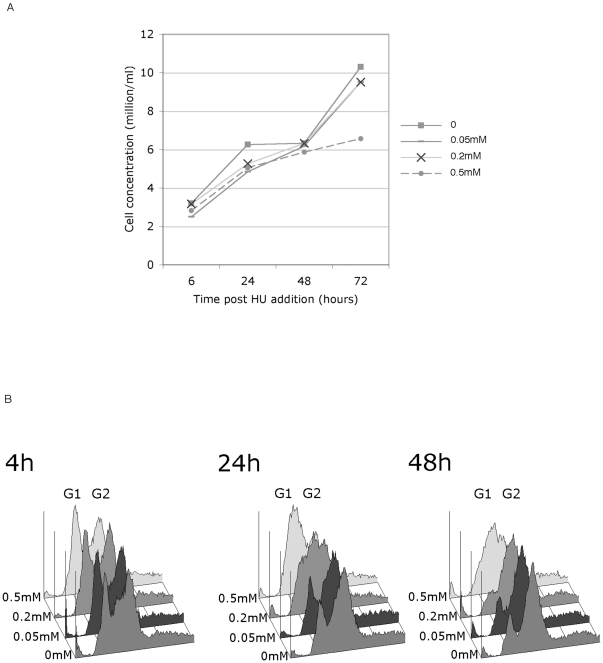
Effect of increasing concentrations of HU on S2 cells. (A)Titration of the proliferation rate of S2 cells treated with different levels of HU. 0 HU (□), 0.05 mM HU (-), 0.2 mM HU (X) and 0.5 mM HU (•) as shown were added to log phase S2 cells and the proliferation rate of the cells was determined by cell counting at 6, 24, 48 and 72 h. Consistent results were obtained with several independent experiments and this figure presents data from one representative experiment. (B) FACS analysis showing the effect of titrating HU into Drosophila S2 cells. Log phase S2 cells were either left untreated or 0.05 mM HU, 0.2 mM HU and 0.5 mM HU as shown (front to back) were added at time = 0 and the cell cycle state of the cells was determined by FACS at 4, 24, and 48 h. Consistent results were obtained with several independent experiments and this figure presents data from the same representative experiment as Figure 4A.

0, 0.05, 0.2 and 0.5 mM HU were added to dividing S2 cells. After 24, 48 and 72 h cell proliferation was measured by cell count, and the effect on the cell cycle was measured by FACS. Figure 4A shows that while 0.05 and 0.2 mM HU have no permanent effect on the cell growth of S2 cells, 0.5 mM appears to significantly affect the doubling time even after 72 hours. Similarly FACS analysis (Figure 4B) suggests that after 48 h the cell cycle profiles of cells treated with 0.05 and 0.2 mM have largely returned to normal, whereas cells treated with 0.5 mM still appear to be largely retained in the S phase. It was therefore decided to use 0.2 mM HU as this clearly had a temporary effect (see 24 h FACS profile) but no long term effect on the cells. Coincidentally the same concentration was also used by Ge et al [11] in human cells.

#### Challenge of depleted and non-depleted cells with HU

0.2 mM HU was added to MCM3, MCM5 and MCM6 depleted and mock treated cells 4 days after the RNAi treatment – this corresponds to a time when >95% MCM protein had been depleted in all cases. The levels of the MCM proteins remain low throughout the rest of the time course of the experiment [10]. The cells were assayed for their proliferation 24, 48 and 72 hours later. For this experiment we did not analyse MCM7 depleted cells since they were already too badly affected to allow meaningful measurements of the additional effect of HU.

**Figure 5:**
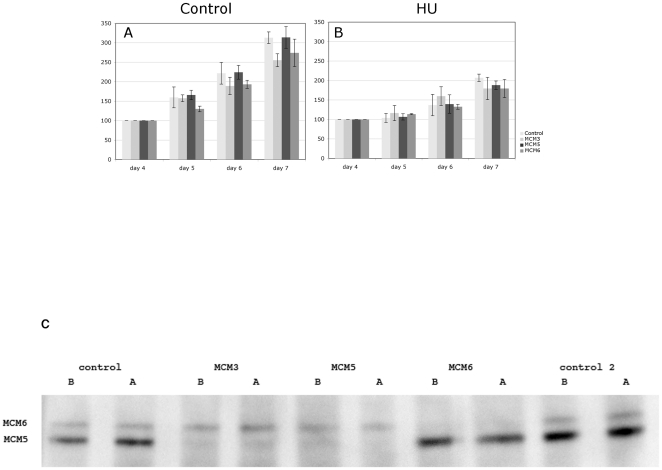
Relative growth rates of control and MCM deplete cells in the absence (5A) and presence (5B) of 0.2 mM HU. These data were derived as explained in Materials and Methods and represents the average of 4 independent experiments. 5C demonstrates that there is no recovery of MCM levels during the time course of the experiment. The levels of MCM5 and MCM6 were measured either 24 h after HU treatment (5 days after dsRNA treatment) – B, or HU was washed out by the addition of new media after 24 h of HU treatment, and the cells were left to grow for a further 2 days - A. The dsRNAs used for the treatment were either against 2 different controls or MCM3, MCM5 or MCM6, as indicated at the top of the figure. The amounts loaded in each lane were checked by ponceau staining of the gel prior to development but the MCM5 and 6 also serve as internal controls for each other.

Figure 5 shows the averaged results from four independent analyses. Figure 5A shows the proliferation profiles of cells which were not treated with HU. Figure 5B shows the same cells with HU added on day 4. In all cases the presence of HU caused a decrease in the growth rate of the cells. We could not detect a significant decrease in the viability of the cells that had lower levels of MCM3, MCM5 and MCM6 as compared to the mock treated cells at 24 and 48 hours after HU addition. On day 7 (after 1 complete division in the presence of HU) there was no significant difference in the MCM depleted cells as compared to the control. (Note that comparable variation was observed when the equivalent sets of cells were allowed to proliferate in the absence of HU treatment.)

An analysis of the cell cycle profile of the cells by FACS failed to show significant additional effects of the removal of MCM proteins in the presence of HU when compared to mock treated cells (data not shown).

We repeated the experiment but instead of leaving the HU in the medium washed it out of the cells after 24 h in case the continuing presence of HU affected the results. In this case we saw no significant effects of MCM depletion on cell growth or cell cycle profile after HU removal (not shown). We also confirmed that the removal of HU (and therefore any dsRNA remaining in the media) did not cause any recovery in the levels of the depleted MCM proteins over the time course of the experiment (Figure 5C).

**Figure 6:**
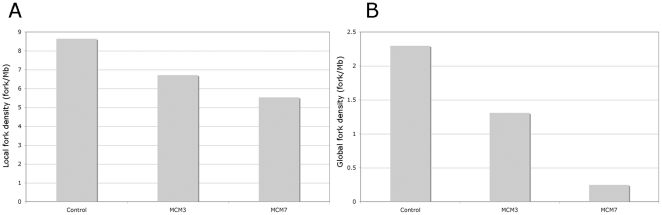
Relative local and global fork densities of MCM depleted and control cells when challenged with 0.2 mM HU as measured using molecular combing. The data were derived as explained in materials and methods. Please also see Figure S2.

We analysed global and local origin densities and rates of fork movement by molecular combing. As seen in Figure 6 and Figure S2 the presence of HU has a small effect (∼20%) on the local fork density and a slightly larger effect (∼40%) on the global fork density in MCM3 depleted cells. This figure also shows that treatment of MCM7 depleted cells with HU had minimal additional effects on fork densities over that seen by the depletion alone. Again in neither case was a significant change seen in the rate of fork movement (see observed BrdU track lengths in Figure S2).

### Reduction of MCM 2-6 in S2 cells has minimal effects on the levels of DNA damage observed in an unperturbed S phase

Ibarra et al reported that depletion of MCM3 caused an increase in damage observed in the absence of replication interference. To test whether this was observed in S2 cells we prepared chromatin from mock and MCM depleted cells 5 days after dsRNA treatment. At this point the cells will have been through 2–3 divisions in the absence MCM proteins which should allow time for damage to develope.

**Figure 7:**
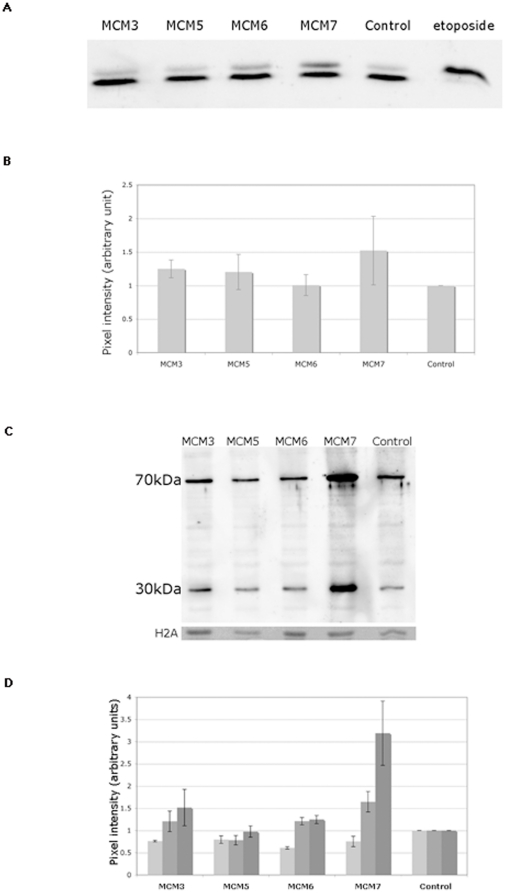
Analysis of the effects of MCM depletion on DNA damage in normally dividing S2 cells. (A) Sample western blot demonstrating the detection of phosphorylated H2AvD as a measure of DNA damage in control and MCM depleted S2 cells. Phosphorylated H2AvD was measured in chromatin prepared from S2 cells at day 4. In all cases the order of loading is MCM3 deplete, MCM5 deplete, MCM6 deplete, MCM7 deplete, TTC4 deplete (control), etop (positive control from etoposide treated cells). Phosphorylated H2AvD is the top band and the loading control is the bottom band - a cross reactivity with H2A. This shows some variability between blots but is consistent within a blot. Loading was also checked by comparison with Ponceau red staining of the blot before development (data not shown). (B) Quantitation of phosphorylated H2AvD as a measure of DNA damage in control and MCM depleted S2 cells on day 5. These data were obtained by quantitation of western blots as shown in (A). The etoposide lane was not quantitated as it was only present to allow the correct identification of phosphorylated H2AvD. (C) Sample western blot demonstrating the detection of chromatin bound RPA as a measure of the level of ssDNA in control and MCM depleted S2 cells. RPA binding was assessed in chromatin prepared from S2 cells at day 5. In all cases the order of loading is MCM3 deplete, MCM5 deplete, MCM6 deplete, MCM7 deplete, TTC4 deplete (control). The 70 and 30 kDa bands of RPA are as shown. The loading control is H2a shown at the bottom of the gel visualized by with Ponceau red staining of the blot before development. (D) Quantitation of RPA binding data for the MCM and control depleted S2 cells as shown on day 3, 4 and 5 (light, medium and dark grey respectively for each condition). These were obtained by quantitation of the 70 and 30 kDa RPA bands from western blots as shown in (C).

To test for ds breaks we used antibodies against phosphorylated Drosophila H2AvD (an H2AX homologue). Figure 7A and 7B shows the results of this analysis. We see a small increase in the level of H2AvD phosphorylation for some of the MCM depleted cells. Again the level of this is variable between experiments in some cases with no additional damage being seen. In MCM7 dsRNA treated cells a much larger increase in H2AvD phosphorylation was observed which is consistent with the marked decrease in proliferation that we see in these cells by this time. It should be noted that despite this small increase in damage for the other MCM deplete lines no significant changes in proliferation and cell growth are observed at this stage.

The presence of ssDNA regions were analysed by determining the level of the RPA 30 kDa and 70 kDa subunits binding to the chromatin. Figure 7C and 7D shows the results of such an analysis 3, 4 and 5 days after the addition of dsRNA. A small increase was seen for MCM3. No significant increases were seen for MCM5 and MCM6. Again a large increase is seen with MCM7, however conclusions about the exact extent of this analysis are complicated by the fact that RPA binding is increased in S phase and MCM7 depleted cells show a higher proportion of S phase cells.

### Reduction of MCM 2-6 in S2 cells has minimal effects on the levels of DNA damage resulting from HU treatment

**Figure 8:**
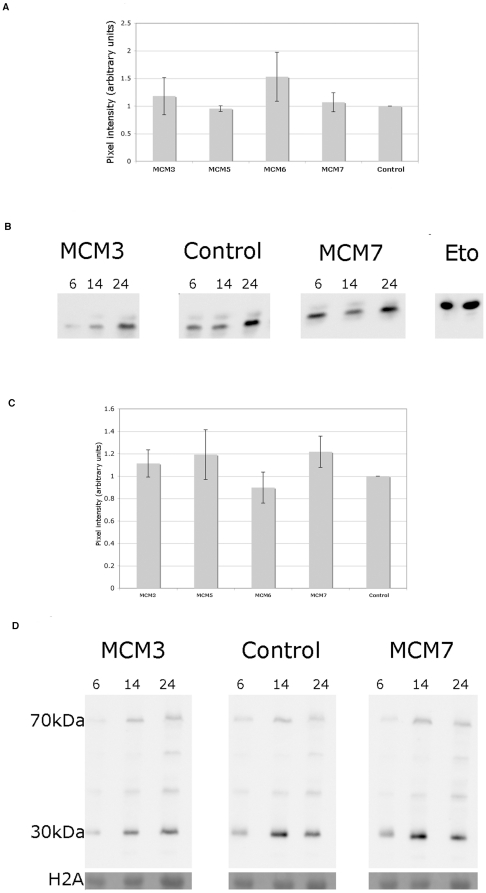
Analysis of the effects of MCM depletion on DNA damage in dividing S2 cells challenged with HU. (A) Quantitation of phosphorylated H2AvD as a measure of DNA damage in control and MCM depleted S2 cells after HU treatment. These data were derived by the quantitation of the levels of phosphorylated H2AvD in chromatin 48 h after HU treatment as seen on western blots equivalent to those shown in Figure 7A. (B) Time course of change in phosphorylated H2AvD levels in chromatin for MCM3, control and MCM7 depleted S2 cells after HU treatment. In each case samples were taken at 6 h, 14 h and 24 h. The last 2 lanes are etoposide treated cell controls and as above the top band is the phosphorylated H2AvD and the bottom, the loading control H2A. (C) Quantitation of chromatin bound RPA in control and MCM depleted S2 cells after HU treatment. These were derived by the quantitation of the levels of the 70 and 30 kDa subunits of RPA in chromatin 48 h after HU treatment from western blots equivalent to those shown in Figure 7C. (D) Time course of change in RPA levels on chromatin for MCM3, control and MCM7 depleted S2 cells. In each case samples were taken at 6 h, 14 h and 24 h. The loading control is H2A shown at the bottom of the gel visualized by with Ponceau red staining of the blot before development.

It was possible that an increase in DNA damage was only observed after replication interference. We therefore carried out chromatin extractions on mock and MCM depleted cells 48 h after 0.2 mM HU treatment. The results from these analyses are shown in Figure 8. DNA damage as measured by H2AvD phosphorylation levels (Figure 8A) was slightly higher for MCM3, and more noticeably for MCM6 at this time point however, as for the experiments carried out in the absence of HU, the level of this is variable between experiments and in some cases no additional damage is seen. In this case even MCM7 depletion had no apparent effect at this time point. In case the damage occurred earlier the cells were also checked at 6 h, 14 h and 24 h after the addition of HU. As can be seen in Figure 8B there were no notable differences in the levels of H2AvD phosphorylation at any of these time points.

Analysis of RPA binding after 24–48 h (Figure 8C) showed slight increases for MCM5 and MCM7 but again these increases were not consistently seen. A time course of RPA binding (Figure 8D) shows that RPA binding is increased at early time points (6 h and 14 h) in the control as well as the MCM3 and MCM7 depleted cells (likely due to the increased number of cells in S phase due to the effect of HU treatment). At later time points, consistent with what was observed previously, no significant differences are seen.

## Discussion

The data presented in this paper allow us to draw several conclusions about the effects of depletion of MCM proteins in Drosophila S2 cells:

### MCM2-6 depletion in the unperturbed cell cycle

These studies confirm our earlier analysis which suggested that depletion of MCM2-6 proteins has no measurable effect on the efficiency of DNA replication in S2 cells. We had already shown that the depletion of MCM2-6 proteins from S2 cells by >95% had no apparent effect on the viability, cell cycling or chromatin association of PCNA. This suggested that there was not a significant effect on DNA replication. However we were concerned that these techniques were not sensitive enough to detect subtle changes that might be caused by the depletion. Therefore in this study we used two techniques -BrdU incorporation and molecular combing – to allow us to detect changes that were not rate limiting to cell growth and viability.

Again we could not detect significant changes in BrdU incorporation, rate of fork movement, or fork density in MCM depleted cells. This leads us to suggest more strongly that the loss of significant amounts of the MCM2-6 proteins from S2 cells has no effect on replication in those cells. This is consistent with previously published data from Xenopus extracts (9), Hela cells for the depletion of MCM3 [12] and U20S cells for the depletion of MCM5 [11] proteins.

In Hela cells although the depletion of MCM3 does not affect cell viability, fork density or fork movement it does produce an apparent increase in the number of ds breaks and ss DNA regions as measured by the chromatin binding of phosphorylated H2AX and RPA respectively. This is much less apparent in Drosophila S2 cells depleted of MCM3, MCM5 or MCM6. Although after averaging several experiments some slight differences were seen, these were not seen consistently in each experiment leading us to question the significance of the differences. This is not due to the MCM/DNA ratio being higher in S2 cells. Quantitative analysis suggests that S2 cells have approximately 2×10^4^ molecules of each MCM per cell compared to transformed human cells which are reported to have 1.5–2.5×10^6^ molecules per cell [15]. Allowing for genome size S2 cells therefore have a 5–10× lower MCM/DNA ratio, more comparable with that reported in non transformed human cells. In addition the S phase lengths are comparable between the two types of cells. Perhaps it is due to a difference between species (Drosophila vs human). It may also be related to the different methodology used for dsRNA interference – in Drosophila the cells do not need to be treated with transfection agents to allow them to take up the RNA. This potentially leaves them less stressed by the procedure itself. Alternatively Hela cells are tumour derived while S2 cells are not and this may also lead to some differences in the response of the cells to replication perturbation similar to that reported for cdt1 overexpression in Hela cells vs fibroblasts [14].

### MCM2-6 depletion and replication interference

The depletion of MCM5 in U2OS cells or MCM3 in Hela cells has been reported to cause sensitisation of cells to replication interference by HU or aphidicolin. This is seen as a decrease in cell proliferation and an increase in observed DNA damage. S2 cells treated with HU do not apparently show the same degree of change in either of these parameters.

Figure 5 shows that as an average of four individual experiments there are no significant decreases in proliferation of MCM3, MCM5 and MCM6 depleted cells at 24 or 48 h after HU treatment. At 72 h there may be some slight effect, however this is not consistently seen in each individual experiment so the significance of these results is unclear. The level of the decreases in proliferation (5–10%) are less than those reported by Ge et al [11] using similar assays for MCM5 depleted U2OS cells treated with 0.2 mM HU (12.5%) and 0.5 mM HU(17%). Larger effects were observed on aphidicolin treatment of MCM3 depleted Hela cells [12], although the maximum effect was observed after 5 days of HU treatment in this case. Ge et al [11] were also able to see larger effects using a different assay involving focus formation in agar although in this case much higher concentrations of HU were used which also had rather severe effects on undepleted cells. Consistent with the reported studies in human cells we did see relatively lower fork densities by combing after HU treatment. These changes were smaller than those reported for human cells which may explain why the observed effects on viability and replication were less prominent. As would be expected the observed differences resulted from an increase in the fork density in untreated cells but no change in the depleted cells.

Replication challenge of MCM3 depleted Hela cells also shows significantly increased levels of ds breaks and ssDNA [12]. MCM5 depleted U2OS cells challenged with HU show some stress related responses: increased levels of chromatin-bound PCNA, ubiquitinated PCNA, RPA, and DNA polymerase delta and phosphorylation p53 on ser15, but not others: changes in the levels of phospho-Chk1, phospho-Rad17, or phospho-H2AX [11]. In S2 cells, although MCM 2–6 depletion does not seem to significantly affect cell viability, we do see some small changes in phospho-H2AvD levels on replication interference. The same is also true for RPA binding. As for the experiments carried out in the absence of HU treatment the precise reason for the differences observed between the different cell types therefore remains unclear, but again may be due to species, cell type or methodology differences.

### Cellular effects of MCM7 depletion

The data presented in this paper also further confirms that S2 cells appear to have additional requirements for MCM7 as compared to the other MCM proteins. We had previously shown that depletion of MCM7 caused severe effects on the proliferation and cell cycle profile of S2 cells. We have now shown that depletion of MCM7, in the absence of replication interference, produces a decrease in DNA synthesis, a decrease in the number of active replication forks (either through a decrease in origin firing or early fork stalling) and an increase in DNA damage. The decrease in replication was not obvious from our earlier studies where we used chromatin binding of PCNA as a measure of ongoing replication. This is likely to be due to a combination of an increased number of MCM7 cells in S phase and PCNA loading as part of the damage response. The effects are not due to a more efficient depletion of MCM7 than the other MCM proteins, as a less efficient depletion of MCM7 produces similar effects. It is also not due to lower levels of MCM7 in cells since in S2 cells protein levels are comparable for each of MCM2-7. The mechanism by which MCM7 functions in this respect is unclear but the negative effects must be related to passage through S phase since treatment of MCM7 depleted cells with HU seems to reduce the level of damage which occurs. In addition since these effects are not seen by depletion of the other MCM proteins it is likely that they are specific to MCM7, although we cannot rule out that it may also be affected by co-depletion of MCM4 with MCM7 [10]. In other systems MCM7 has been suggested to have a role in the checkpoint response to UV damage (human cells [16], [17] and Xenopus extracts – [18]). How this is related to what we observe here will require additional specific studies.

### Conclusion

In S2 cells although the depletion of MCM2-6 causes some small but measurable changes in DNA replication parameters and the levels of damage observed on replication interference these are less than those observed under the corresponding conditions in human cancer cells. In addition these changes have little effect on the viability of S2 cells. This suggests that in S2 cells the excess of MCM2-6 proteins present appears to be greater than that needed for recovery from replication interference possibly suggesting that they may also be required for additional cellular processes (1). By contrast any reduction of MCM7 severely disrupts DNA replication, further strengthening the suggestion that it may play important roles in DNA replication independent of the other members of the MCM complex.

## Materials and Methods

### Antibodies

H2AvD (rabbit anti-histone H2AvD pS137 was obtained from Rockland), anti BrdU was from sigma (B8434). HP labelled anti-rabbit were from Pierce Scientific and HP anti-mouse from Thermo Scientific). Rabbit antibodies against Drosophila RPA were produced using purified Drosophila RPA [19]) as the antigen. MCM3, MCM5 and MCM6 antibodies were as previously reported [20]. Rabbit antibodies against MCM7 were produced at Neosystem (Strasbourg) using hexahis-tagged MCM7 as the antigen.

### Cell culture

S2 cells (originally obtained from the Drosophila Genomics Resource Center) were grown in Schneiders Drosophila medium from Lonza, with 10% Foetal calf serum from Gibco and penicillin streptomycin from sigma.

### DsRNA interference

Primers containing a 5′ T7 RNA polymerase binding site and specific for MCM3, MCM5, MCM6, MCM7 and TTC4 were as previously described [10]. The dsRNA was made using MEGAscript T7 kit (Ambion) as per manufacturers instructions. The dsRNA interference experiment was carried out on S2 cells in exponential growth phase as described [21]. 10 µg of dsRNA was added per 10^6^ cells and the cells were monitored by cell count, FACS analysis and protein blotting over a period of 7 days.

### Measurement of BrdU incorporation by dot Blot

This was carried out largely as previously described [22]. S2 cells at day 5 post RNAi treatment were labelled with BrdU (20 µM) for 1 h. The cells were harvested, resuspended in RSB buffer (10 mM Tris-HCl pH8, 10 mM NaCl, 3 mM MgCl2) at a concentration of 2.5×10^7^ cells/ml, and incubated on ice for 5 min. An equal volume of 0.2% NP-40 in RSB buffer was added followed by incubation in ice for an additional 10 min. The nuclei obtained were pelleted by centrifugation (5000×g for 5 min) and resuspended in 3 ml lysis buffer (200 mM NaCl, 10 mM Tris-HCl pH8, 25 mM EDTA, 1% SDS and 100 µg/ml proteinase K (roche)) overnight at 37°C. The sample was then extracted twice with phenol∶chloroform∶isoamyl alcohol (25∶24∶1). After extraction, an equal volume of isopropanol was added to the aqueous phase and the precipitate was collected by centrifugation for 30 min (16000×g for 30 mins) at 4°C. The DNA was then resuspended in TE buffer (10 mM Tris-HCl pH 8, 1 mM EDTA) and the concentration was measured spectrophotometry.

To denature the sample 5 µl of the DNA solution (at 1 µg/µl) was mixed with 45 µl of NaOH 0.4N, vortexed and incubated for 30 minutes on ice. The solution was neutralised by addition of 50 µl of 1 M Tris-HCl pH8. Then a dilution series of the final solution was spotted on nitrocellulose: Amersham Hybond ECL (G.E). The negative control was DNA extracted from S2 cells (no BrdU labelling). The nitrocellulose membrane was incubated overnight in PBS+1% Tween +1% perfect block (Mo Bi Tech) and incubated with primary (anti BrdU) and secondary antibodies as for protein blotting. Quantification was performed using the Image gauge software on a Fujifilm Life Science LAS-4000 imaging system (Fuji).

### Flow cytometry

Cells were harvested and fixed using 50% ethanol in PBS. Immediately prior to use cells were resuspended in PBS containing 1% glucose, 10 µg/ml RNase,1 mM EDTA,0.5% Triton ×100 and 50 µg/ml propidium iodide to stain DNA. Flow cytometry was carried out on a CYTOMICS 500 (Coulter Beckman) analysis was done using CXP software.

### Cell fractionation (chromatin extraction)

Chromatin fractionation was carried out as described in Crevel et al 2005 [21]. Cells were spun and washed with PBS. They were incubated on ice 10 min, washed twice in PBS 0.5% triton and protease inhibitors (complete, EDTA free, Roche) by resuspension and spinning at 5000×g The final pellet was resuspended in SDS loading buffer at a concentration corresponding to 250 000 cells/µl (5 µl was loaded per well).

### Protein blotting

Proteins from SDS PAGE were blotted onto Amersham Hybond ECL (G.E) and developed with Immobilon Western Chemiluminescent HRP Substrate(Millipore) Visualisation and quantitation were carried out using Fujifilm Life Science LAS-4000 imaging system (Fuji).

### Replication interference analysis

Where appropriate Hydroxyurea (0.2 mM final except when stated) was added to S2 cells at day 4 post addition of dsRNA. The cells were then monitored over a period of 3 days for viability (cell count), cell cycle characteristics (by flow cytometry) and appearance of DNA damage antigens (by western blotting of prepared chromatin against H2AvD and RPA).

In order to get a positive control for H2AvD detection etoposide (10 µM) was added to S2 cells in exponential growth phase and the cells were harvested after (24 h) and chromatin extracted as described above.

### Calculation of growth with HU

Cell count was performed at day 4 5 6 and 7 for controls and HU samples. The cell count obtained at day 4 (before addition of HU) was used as a starting point (value 100%). The growth curve evolving from that point was calculated as a proportion of that initial point. Separate data sets were calculated for the percentage increase or decrease of cells for each depleted MCM and the control with and without HU. The data presented in Figure 4 show the ratio of the percentage growth obtained in the presence of HU to that in its absence for each MCM deplete and the control.

### Combing

To estimate fork progression rate, cells were pulsed with 100 µM BrdU for 30 min. Cells were embedded in agarose plugs and after proteinase K treatment genomic DNA was extracted and “combed” onto cover- slips as described [23]). Coverslips were incubated in 1 M NaOH for 25 min, neutralized in PBS (pH 7.5), and processed for immunofluorescence. BrdU was detected with rat anti-BrdU monoclonal antibody (SeraLab), ssDNA was visualized with an anti-DNA antibody (Chemicon). Images were collected and analyzed with MetaMorph 7 software (Molecular Devices) and analysis of BrdU track vs DNA labeling was performed using the program IDeFIx written by Thierry Gostan (details available from Montpellier DNA Combing Facility).

Global fork density was measured taking the number of BrdU tracks divided by the sum of all fiber lengths. The local fork density was calculated dividing the number of BrdU tracks by the total length of fibers that showed BrdU substitution. In each case only filtered BrdU length was used so any fibres with BrdU at the end of the fibres has been discarded.

## Supporting Information

Figure S1 **Box and whisker plots to show the distribution of the fibre sizes and BrdU tracks obtained by combing which were used for the calculation of the local and global fork densities of control, MCM3, and MCM7 samples in the absence of HU challenge.** For the control, the MCM3 deplete and the MCM7 deplete respectively; the total fibre lengths were 90.7, 29 and 26 Mb, substituted fibre lengths were 10.4, 3.9 and 1.17 Mb and the number of BrdU tracks were 86, 30 and 4.
(TIF)

Figure S2 **Box and whisker plots to show the distribution of the fibre sizes and BrdU tracks obtained by combing which were used for the calculation of the local and global fork densities of control, MCM3, and MCM7 samples in the presence of HU challenge.** For the control, the MCM3 deplete and the MCM7 deplete respectively; the total fibre lengths were 25.7, 29.8 and 32.5 Mb, substituted fibre lengths were 6.8, 5.8 and 1.45 Mb and the number of BrdU tracks were 59, 39 and 8.
(TIF)
