# Supplementary figures and images for: Decreased MCM2-6 in Drosophila S2 Cells Does Not Generate Significant DNA Damage or Cause a Marked Increase in Sensitivity to Replication Interference
Source: PLoS One. 2011 Nov 15;6(11):e27101. doi: 10.1371/journal.pone.0027101 (PMC3216938; doi:10.1371/journal.pone.0027101)

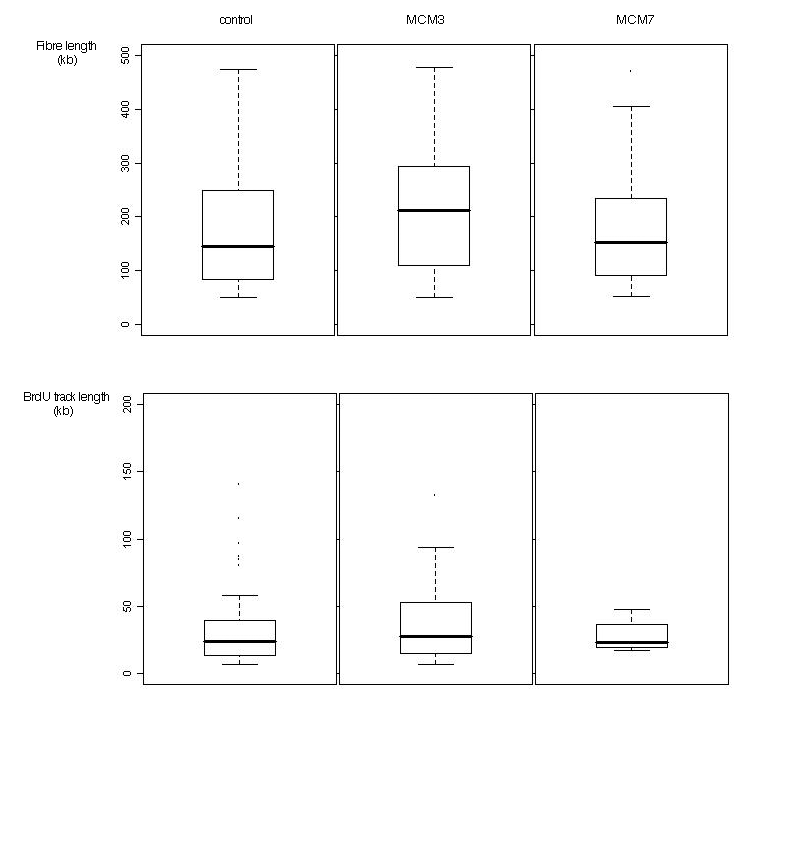

Supplement: Figure S1 — Box and whisker plots to show the distribution of the fibre sizes and BrdU tracks obtained by combing which were used for the calculation of the local and global fork densities of control, MCM3, and MCM7 samples in the absence of HU challenge. For the control, the MCM3 deplete and the MCM7 deplete respectively; the total fibre lengths were 90.7, 29 and 26 Mb, substituted fibre lengths were 10.4, 3.9 and 1.17 Mb and the number of BrdU tracks were 86, 30 and 4. (TIF) [file pone.0027101.s001.tif]

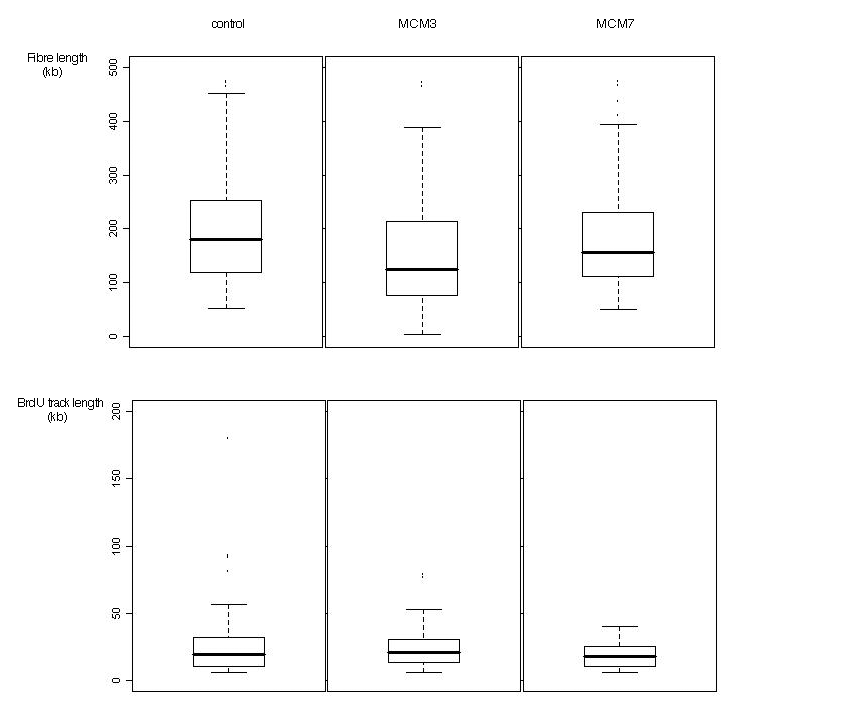

Supplement: Figure S2 — Box and whisker plots to show the distribution of the fibre sizes and BrdU tracks obtained by combing which were used for the calculation of the local and global fork densities of control, MCM3, and MCM7 samples in the presence of HU challenge. For the control, the MCM3 deplete and the MCM7 deplete respectively; the total fibre lengths were 25.7, 29.8 and 32.5 Mb, substituted fibre lengths were 6.8, 5.8 and 1.45 Mb and the number of BrdU tracks were 59, 39 and 8. (TIF) [file pone.0027101.s002.tif]
